# The management of perioperative pain in craniosynostosis repair: a systematic literature review of the current practices and guidelines for the future

**DOI:** 10.1186/s40902-022-00363-5

**Published:** 2022-10-14

**Authors:** Hatan Mortada, Raghad AlKhashan, Nawaf Alhindi, Haifa B. AlWaily, Ghada A. Alsadhan, Saad Alrobaiea, Khalid Arab

**Affiliations:** 1 Department of Surgery, Division of Plastic Surgery, King Saud University Medical City, King Saud University Medical City, Riyadh, Saudi Arabia; 2 Department of Plastic Surgery & Burn Unit, King Saud Medical City, Riyadh, Saudi Arabia; 3 Faculty of Medicine, King Saud University, Riyadh, Saudi Arabia; 4 College of Medicine, King Abdulaziz University, Rabigh, Saudi Arabia; 5 Department of Plastic Surgery and Burn Unit, Security Forces Hospital, Riyadh, Saudi Arabia; 6 Division of Plastic Surgery, Department of Surgery, College of Medicine, King Saud University, Riyadh, Saudi Arabia

**Keywords:** Pain control, Cranial vault reconstruction, Craniosynostosis, Pain management, Cranioplasty

## Abstract

**Background:**

Craniosynostosis is a condition characterized by a premature fusion of one or more cranial sutures. The surgical repair of craniosynostosis causes significant pain for the child. A key focus of craniosynostosis repair is developing effective strategies to manage perioperative pain. This study aimed to review perioperative pain control strategies for craniosynostosis repair systematically.

**Methods:**

Guidelines for reporting systematic reviews and meta-analyses were used in the design of this review. In May 2022, the following databases were used to conduct the literature search: MEDLINE, Cochrane, EMBASE, and Google Scholar. A search was performed using MeSH terms “craniosynostosis,” “pain management,” and “cranioplasty.”

**Results:**

The literature review yielded 718 publications. After applying our inclusion criteria, 17 articles were included, accounting for a total of 893 patients. During the postoperative period, most studies used multimodal analgesia, primarily opioids, and acetaminophen. In the postoperative period, oral ibuprofen was the most commonly used NSAID, rectal codeine, and acetaminophen were the most commonly used weak opioids, and continuous remifentanil infusion was the most commonly used potent opioid.

**Conclusion:**

The authors determined the best pain management options for pediatric patients undergoing cranioplasty by analyzing the most commonly used analgesics. A high-quality clinical trial comparing different types of analgesic combinations would be a valuable addition to the present literature.

## Background

Craniosynostosis is a rare condition characterized by the premature fusion of one or more cranial sutures. The cranium is formed during development via intramembranous ossification, leaving the sutures not fully ossified to allow passage through the birth canal and expand brain growth [1]. Craniosynostosis must be managed early to avoid damaging adverse outcomes, including blindness, abnormalities in skull shape, and developmental impairments of the brain that may significantly affect the child’s quality of life. Craniosynostosis can be corrected through craniotomies and cranioplasties, which are invasive and painful procedures due to the extensive handling of the scalp and periosteum [2].

Currently, there is no standard protocol for managing perioperative pain associated with craniosynostosis repair [3]. Several studies have shown that steroids can be used preoperatively to reduce postoperative pain as a secondary benefit, along with other benefits such as reducing facial edema, reducing postoperative ecchymosis, and improving nausea and vomiting [4–9].

Current recommendations mostly aim to achieve a balanced technique that provides cardiovascular stability by using opioids and volatile agents in addition to relaxants. Remifentanil infusion (0.25–0.5 mcg/kg/min) is also recommended [10–12]. In addition, in case of remifentanil usage for anesthesia maintenance, it is recommended to administer a bolus of morphine or piritramide before the end of the procedure to help manage postoperative pain [13]. However, there is still no clear evidence on a specific intraoperative opioid regimen that provides the maximal benefit to pain management. Kattail et al. found that among patients with non-syndromic craniosynostosis, within the first few days following surgery, a significant number of patients complained of moderate to severe pain, which suggests that pain was poorly treated despite the use of intraoperative opioids in all patients. Subsequently, the authors attributed this finding to the underutilization of non-opioid analgesics [14].

Despite the extensive body of literature exploring the operative treatment of craniosynostosis, there is still a lack of consensus on the optimal perioperative management protocols, including pain control regimens. This might be explained by the lack of verbalization in young children about their pain [3]. In the literature, opioids alone, opioids combined with acetaminophen or non-steroidal anti-inflammatory drugs (NSAIDs), and local nerve blocks have all been described as methods of treating postoperative pain [15]. It has been reported that many attending physicians in pediatric intensive care units (PICUs) use intravenous (IV) dexmedetomidine on a postoperative day one in conjunction with IV acetaminophen to replace morphine. Dexmedetomidine is rarely used postoperatively in pediatric plastic surgery, and current reports focus mostly on cases of pediatric cleft lip and cleft palate. These discrepancies in the available research regarding postoperative pain management in craniosynostosis make it a clinical challenge for plastic and reconstructive surgeons [15–18].

There is a lack of information specifically regarding the current techniques and efficacy of perioperative analgesia for such procedures among craniosynostosis patients [3]. Therefore, this systematic review aimed to compare the literature on perioperative pain management to provide the best evidence-based pain management options for all children undergoing craniosynostosis repair. In addition, clinical outcomes have been reviewed in the literature, recommendations, and administration methods for different perioperative pain management options.

## Methods and materials

### Review of the literature

We conducted this systematic review using the Preferred Reporting Items for Systematic Reviews and Meta-Analyses (PRISMA) guidelines, in accordance with Cochrane review methods [19, 20]. The published literature was searched on MEDLINE, Cochrane, EMBASE, and Google Scholar from inception until May 2022 without specifying a timeframe. Bibliographies of reviewed articles identified additional articles. As part of the literature review, the following terms and keywords were used: (craniosynostosis or cranial vault reconstruction or cranial reconstruction or cranioplasty) and (pain management or analgesia or analgesics or pain control). This study aimed to review and compare literature on perioperative pain management to provide the best evidence-based options for all children undergoing craniosynostosis repair. The proposal was registered to the International Prospective Register of Systematic Reviews (PROSPERO) guidelines (ID number: CRD42022339835) [21].

### Selection of the studies

The following criteria were used to determine inclusion: (1) published studies that are not time-limited, (2) published in English, (3) human studies, (4) reported RCT, (5) prospective/retrospective cohort studies, (6) prospective/retrospective case series, (7) pediatric patients, (8) patients with craniosynostosis, (9) a clear description of pain management protocols, and (10) clinical outcomes of interest were reported.

Among the exclusion criteria were (1) studies published in non-English languages; (2) inappropriate methods (case reports, meta-analysis and systematic reviews, cadaver studies, narrative review, or editorial); (3) non-craniosynostosis patients; (4) animal studies; (5) not providing a complete description of the perioperative pain management protocol; and (6) reporting no findings.

Based on predefined inclusion and exclusion criteria, all abstracts of included studies were screened using the Rayyan search engine [22]. The studies were then included by title and abstract and were divided into two groups, each with two independent reviewers. All selected articles by both groups were reviewed by a fifth independent reviewer to resolve disagreements. Both groups reviewed the full texts of the studies to ensure compliance with inclusion and exclusion criteria.

### Extraction of data

An Excel sheet was created to review the full texts, and the outcome measures were extracted. From the final included studies, data parameters included general parameters (title, author, year of publication, country, study design, total number of patients, number of patients with craniosynostosis), demographics (age in months (SD), number of males and females, race, type of syndrome, type of craniosynostosis, comorbidities, and name of surgical intervention), methods of pain management (name of medications, doses, timing (preoperative, intraoperative, and/or postoperative), complete analgesic protocol, complications, length of hospital stay, and follow up), and name of pain score used to determine the efficacy of pain control, parental satisfaction, and a summary of the significant primary outcomes and clinical recommendations. A disagreement regarding the extraction and screening of data was resolved by two senior independent reviewers. The retrieved data were double-checked to avoid duplication. All articles included in the review were rated according to the level of evidence and grading recommendations of the American Society of Plastic Surgeons [23].

### Assessment of bias

Two reviewers independently assessed the risk of bias using the Newcastle–Ottawa Scale for case–control and cohort studies [24]. With this scale, the risk of bias is assessed in the domains of selection, comparability, and outcomes and is rated up to a maximum of 9. Studies with scores of 0–3 had a high risk of bias, those with scores of 4–6 a moderate risk, and those with scores of 7–9 a low risk. Based on eight components, the methodological quality and synthesis of case series and case report assessment tools are divided into four domains: selection, ascertainment, causation, and reporting [25]. A Cochrane risk-of-bias tool for randomized trials was used for assessing randomized controlled trials for bias [26]. Every study category was rated based on randomization, allocation concealment, participant and employee blinding, observer blinding, incomplete data, and selective reporting.

### Analysis of data

Although a basic descriptive statistical analysis was performed, meta-analysis was not possible due to the heterogeneity of the articles included.

## Results

### Findings from literature

**Fig. 1:**
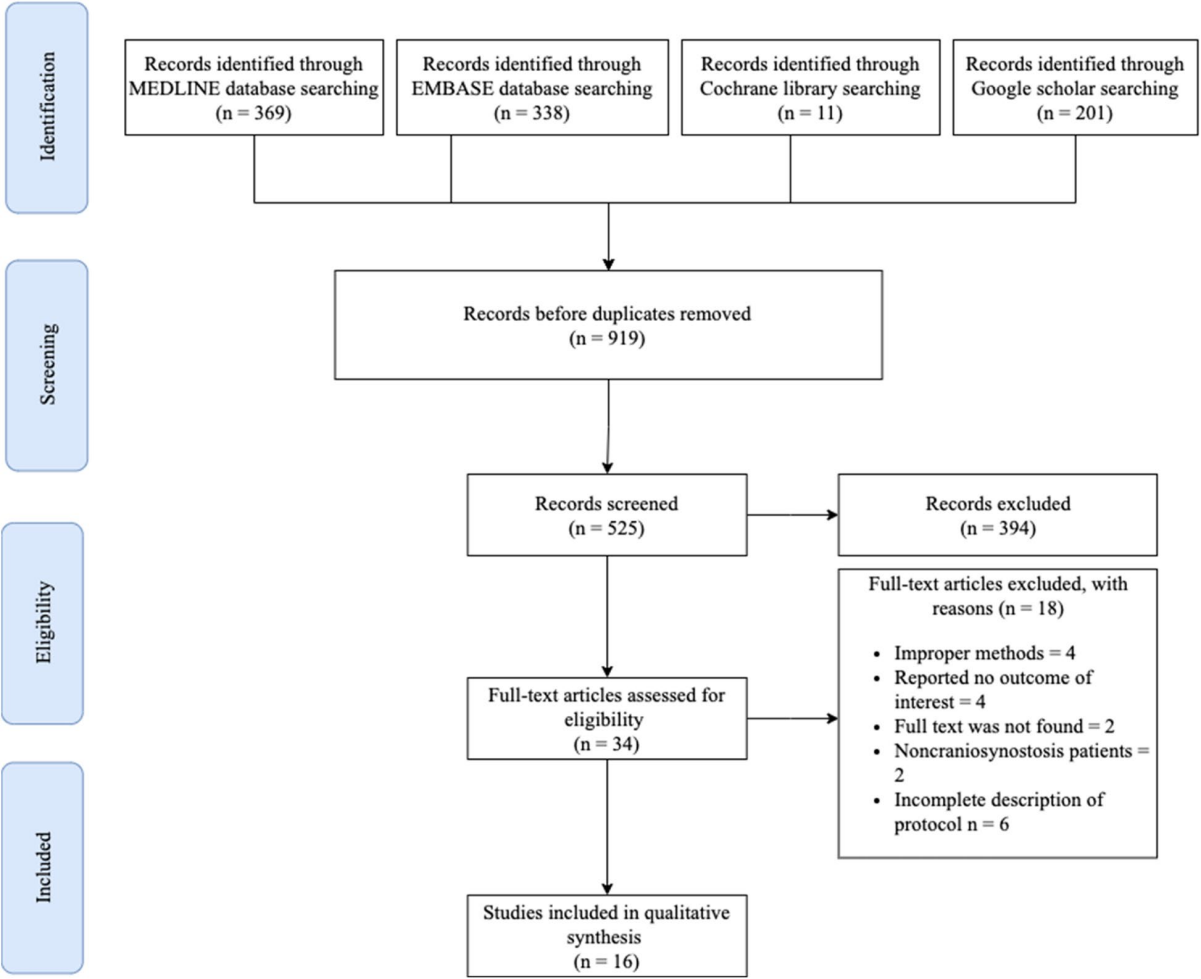
The PRISMA flowchart for systematic review. The process of selecting the included studies

In this systematic review, 919 published articles were found, including 338 articles from EMBASE, 369 articles from MEDLINE, 201 from Google Scholar, and 11 articles from the Cochrane Library. There remained 525 articles for review after removing duplicates. We included 34 articles based on their titles and abstracts in the initial screening. Based on the previously defined exclusion criteria, only 16 articles published between 2000 and 2022 were included (Fig. 1) [2, 10, 14, 27–39]. A total of 18 articles were excluded for the following reasons: improper methods (meta-analysis/systematic review, case reports) *n* = 4, reported no outcomes of interest (*n* = 4), no full text was found (*n* = 2), non-craniosynostosis patients (*n* = 2), and incomplete description of perioperative pain management protocol (*n* = 6). There were three prospective cohort studies, three randomized controlled trials, seven retrospective studies, two case–control studies, and one case series among the included studies. Most studies were conducted in the USA (*n* = 7). Three studies were conducted in Italy, three in the Netherlands, two in France, and one in Canada. The included articles were all on pediatric patients with craniosynostosis who underwent cranioplasty, except for two papers that included other craniotomies. The study included only patients who had undergone cranioplasty. Detailed characteristics of all the included studies are demonstrated in Table 1.

**Table 1:** The characteristics of the studies included and the patients recruited

Author	Study design	Country	Number of patients	Age in months, SD	M/F^a^	Race	Comorbidities	Type of syndrome	Type of craniosynostosis	Level of evidence
Chiaretti [10]	P	Italy	20	3.9	11/8	NA	NA	Crouzon’s (5), Apert’s (1)	Scaphocephaly (8), anterior plagiocephaly (6)	II
Marel [28]	RCT	Netherland	40	2.7	29/11	NA	NA	NA	Trigonocephaly 7, scaphocephaly 20, plagiocephaly 9, brachycephaly 4	I
Warren [31]	R	Canada	71	18	NA	NA	NA	NA	Sagittal 16, coronal 12, metopic 7, other 5	II
Jong [27]	RCT	Netherland	60	6.8	45/15	NA	NA	NA	Trigonocephaly 5, scaphocephaly 8, plagiocephaly 5, other 2	I
Bracho [36]	P	France	32	16	19/13	NA	NA	NA	Trigonocephaly 12, scaphocephaly 7, plagiocephaly 6, brachycephaly 3, pachycephaly 4	II
Bronco [30]	P	Italy	206	3.1	123/86	NA	NA	NA	NA	II
Fearon [35]	RCT	USA	50	52	38/12	white (38), non-white (12)	NA	NA	NA	II
Arts [29]	R	Netherlands	121	3.9	85/36	NA	Atopy (4), viral infection (3), neutropenia (1), facial malformation (2), cardiac (2), pulmonary (2),	Abert (3), Muenke (2)	Scaphocephaly (63), trigonocephaly (36), plagiocephaly (14), brachycephaly (1), syndromal (5), multisutural (2)	II
Cercueil [38]	CC	France	81	11	NA	NA	NA	NA	Trigonocephaly (35), scaphocephaly (31), other (15)	III
Kattail [14]	R	USA	54	21.1	30/24	White (36), black (8), others (10)	NA	Nonsyndromic	NA	II
Tuncer [37]	R	USA	74	30.6	44/30	NA	None	NA	Sagittal (24), metopic (15), unilateral coronal (10), lambdoid (3), multisuture or complex (22)	II
Reddy [32]	R	USA	80	30	47/33	Asian (2), black (20), white (50), other (8)	NA	NA	Single suture (37), double suture (10), triple suture (13), 4 or more (20)	II
Xu [2]	CS	USA	2	20	2/0	NA	Short gut, premature, and enterocolitis, significant anemia and developmental delay	NA	Sagittal and metopic craniosynostosis	IV
Festa [39]	CC	Italy	26	7.8	15/11	NA	NA	NA	Scalp block group: scaphocephaly (6), trigonocephaly (3), right anterior plagiocephaly (2), complex craniosynostosis (2) Control group: scaphocephaly (8), trigonocephaly (2), right anterior plagiocephaly (1), left anterior plagiocephaly (1), complex craniosynostosis (1)	III
Knackstedt [34]	R	USA	78	33.6	34/34	NA	NA	NA	NA	II
Zubovic [33]	R	USA	43	48	5/38	NA	NA	NA	NA	II

*CC* Case control, *CS* Case series, *R* Retrospective cohort, *P* Prospective cohort, *M* Male, *F* Female, *NA* Not available, *USA* United States of America

^a^Gender distribution was based on the total number of patients with craniosynostosis

### An overview of the studies’ characteristics

**Fig. 2:**
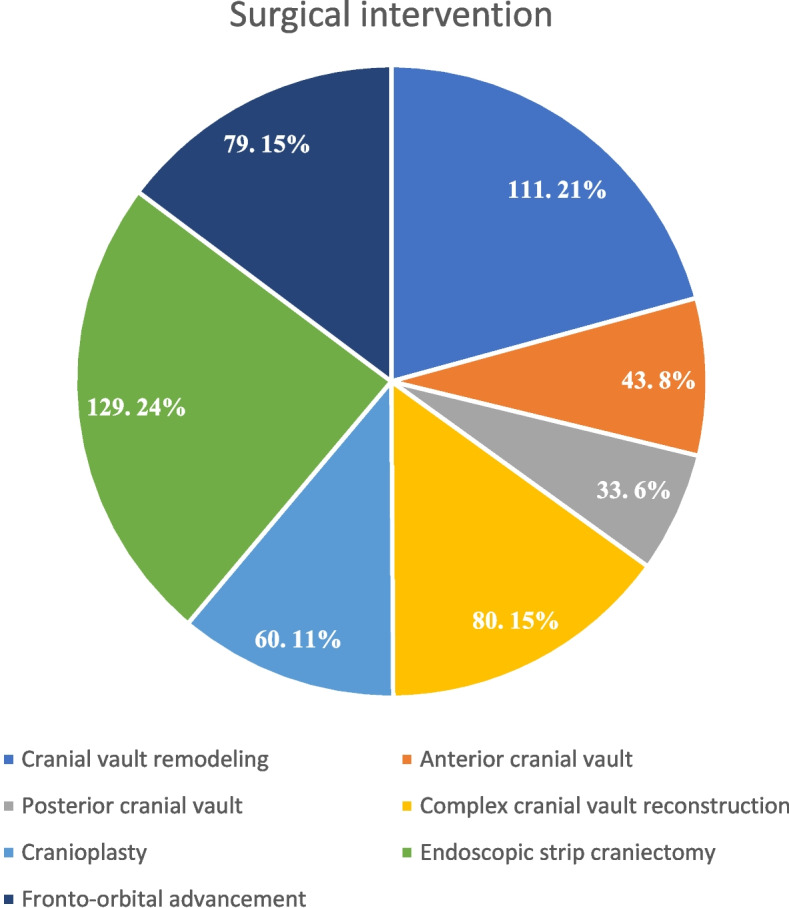
Type of surgery in the included studies

**Fig. 3:**
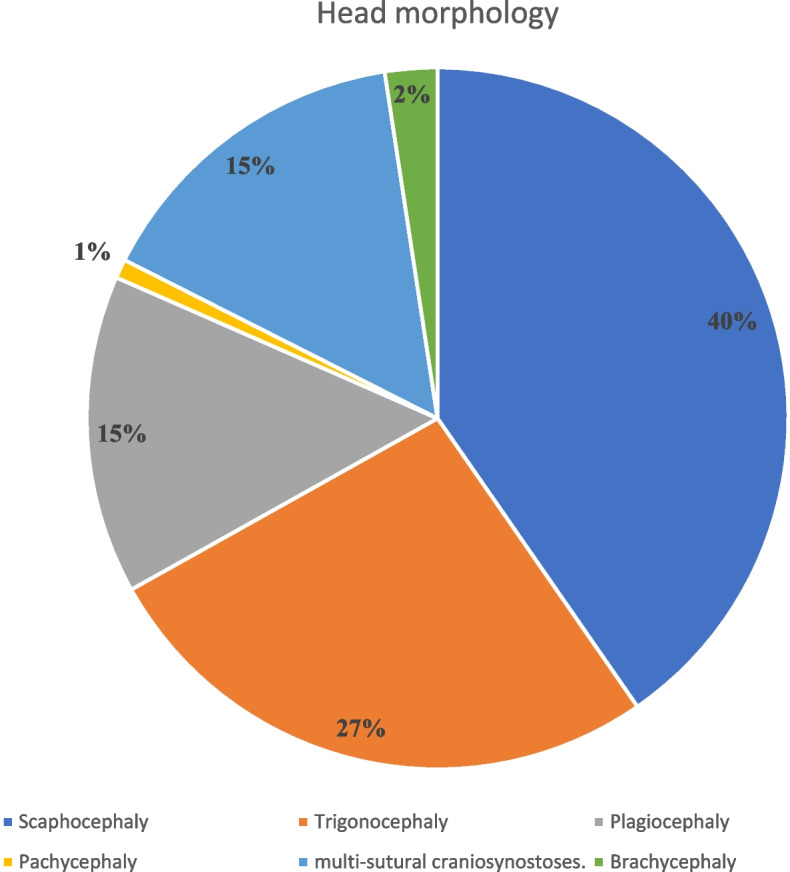
Head shapes of the included patients with craniosynostosis

From all the studies, 1038 patients were reviewed. There were a total of 848 patients with craniosynostosis. The age of the patients ranged from 3.1 to 55 months. The majority of included patients were males (*n* = 527/848, 62.14%); however, gender was not mentioned in two articles [31, 38]. Race was only mentioned in three studies [14, 32, 35], which showed the majority of patients were White (*n* = 124), Black (*n* = 28), and Asian (*n* = 2). There were only 11 patients with syndromic craniosynostosis, 4 with Apert syndrome, 2 with Muenke syndrome, and 5 with Crouzon’s syndrome. There were 184 cases of scaphocephaly, 121 trigonocephaly, 67 plagiocephaly, 11 brachycephaly, 4 pachycephaly, and 69 multi-sutural craniosynostoses (Fig. 2). The type of craniosynostosis was not mentioned in 5 studies [14, 30, 33–35]. Among the included patients, the majority underwent cranial vault remodeling (*n* = 111), followed by endoscopic strip craniectomy (*n* = 129), and followed by fronto-orbital advancement (*n* = 78). Figure 3 illustrates the different surgical interventions among the included patients. For greater clarity and comprehension, the authors separated analgesia delivery methods into two categories: intraoperative and postoperative.

### Intraoperative analgesia

A total of 441 (52%) patients were included in seven articles describing the complete intraoperative analgesic protocol [14, 32, 34, 36, 38, 39]. Reddy et al. reported eighty patients were placed into one of two groups, with 39 receiving intraoperative dexmedetomidine and 41 who did not. Postoperatively, neither group had a difference in opioid requirement or pain score. There was no significant difference between the two groups in terms of the number of days spent in the PICU, overall hospital stay, or duration on a mechanical ventilator. However, in patients given higher doses of intraoperative dexmedetomidine, the use of rescue medications for nausea and vomiting was significantly lower (*p* = 0.017) [32].

A total of two studies used Scalp Nerve Block (SNB) [36, 39]. A study by Bracho et al. reported 32 children undergoing craniosynostosis surgery under general anesthesia with associated levobupivacaine (0.125% 2 mg/kg)/epinephrine (1.25 mg/mL) Scalp Nerve Block (SNB) followed by 15 mg/kg of IV acetaminophen 30–45 min prior to skin closure and then every 6 h. In the surgical ward, nalbuphine was prescribed at 0.2 mg/kg once a CHEOPS score of 8 or an Aono’s four-point scale score greater than 2 was reached. According to the study, the SNB technique offers many advantages, including the ability to limit injections to specific nerves, reduce the volume required of local anesthetic, provide better hemodynamic stability at skin incision and closure, and reduce opioid use and dosage. For intraoperative analgesia, five more studies were found. One study used IV morphine and acetaminophen, another IV opioid alone, and one followed enhanced recovery after surgery (ERAS), which involves hemoglobin optimization, cell-saver technology, tranexamic acid, and intraoperative interventions, such as gabapentin and local anesthetic, fluid titrations postoperatively, and transfusion protocols. Scheduled acetaminophen, ibuprofen, or ketorolac are the preferred analgesics, and dexmedetomidine is used with opioids only when breakthrough pain occurs. Analgesic protocols for each study are shown in Table 2.

**Table 2:** An overview of characteristics and analgesic protocols for intraoperative pain management

Author	Surgical intervention	Name of pain management drug	Dose of each drug	Complete analgesic protocol	Complications	LOS	Clinical recommendations	Significant outcomes
Bracho [36]	NA	Levobupivacaine, acetaminophen, morphine, and nalbuphine	Acetaminophen (15 mg/ kg IV*), morphine (0.02 mg/kg)	An acetaminophen dose 30–45 min before closing skin, then every 6 h (depending on postoperative pain score addition analgesic administered (morphine))	Sedation, N/V*	NA*	Patients undergoing craniosynostosis surgery can benefit from SNB* technique to complement analgesia	NA
Bronco [30]	NA	Acetaminophen, ibuprofen, codeine + acetaminophen, tramadol, remifentanil, morphine, fentanyl.	Remifentanil (0.2 µg/kg), fentanyl (4 µg/kg)	Analgesic therapy given after extubation. Analgesics were not administered to 14 children (7%) during the first day after surgery, and another 41 (20%) during the second day.	Delirium, sedation, respiratory depression, N/V	NA	After a major craniotomy, children receiving multimodal analgesia experienced little or no pain.	NA
Cercueil [38]	NA	Morphine, acetaminophen, nalbuphine	Morphine (0.1–0.2 mg/kg), acetaminophen (15 mg/kg)	Both administered IV before the end of surgery, postoperative morphine in the recovery room until FLACC* is 3/10. Following recovery room discharge, administration of IV acetaminophen combined with oral morphine (or IV nalbuphine as rescue if necessary).	None	NA	Confirm data by prospective studies	Patients in the local anesthetic infiltration group had a modest reduction in morphine use, but no differences in pain scores compared to the SNB group.
Kattail [14]	Open craniosynostosis repair	Acetaminophen, Intravenous ketorolac, Dexmedetomidine, Fentanyl, Morphine, Hydromorphone, Sufentanil, remifentanil	Acetaminophen 12.5 mg/kg every 4 h,	Intraoperative: All patients received IV opioids, in addition to: fentanyl, hydromorphone and fentanyl, hydromorphone, fentanyl and morphine, morphine, sufentanil, or fentanyl and remifentanil. Postoperative: IV PCA*. Fentanyl, morphine, and hydromorphone were the opioids administered to patients who received PCA. On the first or second day postoperatively, the majority of patients were able to transition to enteral formulation. Each patient receiving enteral opioids was prescribed oxycodone at a dose of 0.1 mg/kg (0.01 mg/kg), which was administered every 4 h. Scheduled ‘‘around the clock’’ or ‘‘as needed’’ dosing of oxycodone basis. All patients received acetaminophen at a dose of 12.5 mg/kg every 4 h. Route of administration of acetaminophen varied, mostly orally, some rectally, and a few IV. Six patients received IV ketorolac, and 4 received Dexmedetomidine.	Emesis	3.7 (1.9)	Pain control, emesis reduction, and LOS* reduction can be achieved through the implementation of ERAS protocols and the use of non-opioid analgesics after surgery.	According to a multivariable linear regression model, age (*P* = 0.006), weight (*P* = 0.009), and postoperative day of transition from IV to enteral opioids were independent predictors of overall hospital stay length (*P* < 0.001).
Reddy [32]	Complex cranial vault reconstruction	Dexmedetomidine, morphine		Both cohorts received morphine, one cohort (*n* = 39) also received dexmedetomidine	NA	Control = 4.2 ± 1.0, dexmedetomidine cohort = 4.0 ± 0.8	There is still a need for further investigation into the relationship between dexmedetomidine and lower antiemetics use.	Ondansetron doses and intraoperative dexmedetomidine dosages (*P* = 0.017).
Festa [39]	Mininvasive procedure: 6 in ST group, 7 in SB group Open remodeling: 7 in ST group, 6 in SB group	Levobupivacaine, acetaminophen, tramadol, ketoprofene.	Levobupivacaine 0.125% (total dose 2 mg/kg), acetaminophen 10 mg/kg every 8 h, tramadol 1 mg/kg every 12 h, ketoprofene 1 mg/kg every 8 h	All patients in this study received acetaminophen at a dose of 10 mg/kg every 8 h. If there was still pain, the attending physician delivered tramadol at 1 mg/kg every 12 h or ketoprofene 1 mg/kg every 8 h. For the scalp block group only: a targeted infiltration of 0.75–2 ml of local anesthetic solution was administered at multiple sites via a 23G needle.	None	PICU = scalp block group: 21.1—control group: 18.1	A multimodal approach consisting of SNB + acetaminophen was effective for immediate postoperative pain control in pediatric patients aged less than 2 years who underwent cranioplasty for craniosynostosis.	- Weak evidence in SNB group which showed a longer LOS (*P* = 0.04) - Strong evidence in SNB patients which showed earlier oral feeding for both clear fluid and milk (*P* = 0.001)
Knackstedt [34]	Fronto-orbital advancement	Dexmedetomidine, acetaminophen, Ketorolac, ibuprofen, oxycodone and morphine	Acetaminophen (15 mg/kg IV) q6h. Ketorolac (0.25 mg/kg IV) q6h, ibuprofen (10 mg/kg PO) q6h, oxycodone (0.05 mg/kg PO) q6h prn, morphine (0.05 mg/kg) q4h prn	Intraoperative: At closure, dexmedetomidine drips are started and continued postoperatively. During postoperative recovery, the drip is titrated to effect and maintained until the first morning following surgery. Postoperative: Every 6 h, 15 mg/kg of acetaminophen is administered intravenously. Every 6 h, ketorolac or ibuprofen are administered. Nurses have the discretion to choose one over the other. Oxycodone and morphine are available and may be given as needed.	NA	ERAS group: 2.3 control group: 3.6	By using the ERAS approach, the overall as well as the intraoperative allogenic blood transfusion rates were reduced, narcotics were used less, and hospital stays were shorter.	- Patients in the ERAS protocol had a decreased overall LOS (*P* = 0.02) - Fewer patients in the ERAS protocol required intraoperative blood transfusion (*P* < 0.0001) - From the ERAS protocol patients who required morphine or PO narcotics, fewer doses were needed (*P* = 0.0005, *P* = 0.007, respectively).

^*^NA Not available, *IV* Intravenous, *N/V* Nausea/vomiting, *SNB* Scalp nerve block, *FLACC *Face, Legs, Activity, Cry, Consolability, *PCA* Parent/patient-controlled analgesia, *LOS* Length of stay

### Postoperative analgesia

In total, nine articles describing the complete postoperative analgesic protocol were identified [2, 10, 27–29, 31, 33, 35, 37], including 407 (47.9%) patients. In a prospective randomized controlled trial of 40 craniosynostosis patients, Van der Marel et al. compared oral acetaminophen versus rectal acetaminophen. Each patient underwent preoperative SNB using bupivacaine and epinephrine. Those receiving rectal acetaminophen had significantly higher plasma levels of the drug. In addition, patients receiving oral acetaminophen scored significantly higher on the COMFORT and VAS scales (P1⁄40.02 and P1⁄40.04, respectively). However, plasma acetaminophen concentrations did not significantly correlate with pain scores [28]. Another study by Tuncer et al. showed that using 10 mg/kg ibuprofen; 0.25 mg/kg IV ketorolac postoperatively was associated with shorter hospital stay (*P* < 0.05) and less morphine for pain control [37]. The use of narcotics in craniosynostosis repair surgery was described by Bronco et al. in a multicenter study of 90 patients. Postoperatively, oral ibuprofen was the most commonly used NSAID, rectal codeine in association with acetaminophen was the most commonly used weak opioid, and continuous infusion of remifentanil was the most widely used potent opioid [30]. In another study, Chiaretti et al. examined 20 patients using remifentanil prospectively [10]. The use of opioids in 54 pediatric patients undergoing primary open craniosynostosis repair was reported by Kattail et al. [14]. In the intravenous parent/patient-controlled analgesia (IV PCA) protocol, fentanyl (51%), morphine (41.2%), and hydromorphone (7.8%) were administered intravenously. De jong et al. compared the effects of the “M” technique massage with or without mandarin oil compared to standard postoperative care on infants [27]. A study by Xu et al. reported the use of dexmedetomidine as an adjunct to IV acetaminophen and as a substitute for morphine in craniosynostosis repair [2]. One study reported the use of continuous morphine infusion [31]. Another study reported the use of oxycodone suspension as the only opioid prescribed at discharge [33]. Lastly, one study described postoperative management as prescribing scheduled IV acetaminophen and Ketorolac or ibuprofen [35]. Table 3 provides a detailed description of the postoperative analgesic protocol.

**Table 3:** An overview of characteristics and analgesic protocols for postoperative pain management

Author	Surgical intervention	Name of pain management drug	Dose of each drug	Complete analgesic protocol	Complications	LOS	Clinical recommendations	Significant outcomes
Jong [27]	Cranioplasty	Isoflurane, Sevoflurane, Iso- and Sevoflurane, Acetaminophen IV or supp, Morphine IV, Fentanyl IV, Sufentanyl IV, Remifentanil IV, Piritramide IV, Propofol IV, Midazolam IV.	Single dose	1- ‘M’ technique massage with carrier oil only, i.e., almond oil 2- ‘M’ technique massage with mandarin 1% in carrier oil	NA	NA	‘M’ technique massage can be used as a comforting mechanism	NA
Fearon [35]	All cranial vault remodeling procedures	Oral ibuprofen, acetaminophen, intravenous ketorolac	Intravenous ketorolac 0.5 mg, oral Ibuprofen 10 mg, acetaminophen 15 mg/kg.	Patients in the control group were given oral ibuprofen and acetaminophen only, while the treatment group was given IV ketorolac and acetaminophen only. Neither group received any postoperative narcotics and thresholds for the medications were determined by standard pediatric nursing assessments for discomfort.	Postoperative nausea and vomiting	2	Administer all nonnarcotic pain drugs IV	IV administation decreased severe vomiting significantly (*P* value < 0.001) compared to oral
Arts [29]	Endoscopic strip craniectomy	Acetaminophen, low-dose morphine	Acetaminophen 80 mg/kg/d, low dose morphine 5–40 mg/kg/h	Mainly acetaminophen, Morphine was started, when required, at 5 lg/kg/h and increased to a maximum of 40 mg/kg/h depending on the CHIPPS score.	Decline in hemoglobin and hematocri, blood loss	NA	NA	NA
Tuncer [37]	Anterior cranial vault, Posterior cranial vault remodeling	Ketorolac, ibuprofen, oxycodone, morphine	Morphine, Acetaminophen. Before skin incision, either a scalp block or local anesthetic infiltration was performed with 1 mL/kg of 0.25% levobupivacain, associated with epinephrine (0.01 mg/mL) in case of infiltration	10 mg/kg ibuprofen; 0.25 mg/kg IV ketorolac	The discharge hemoglobin is lower in the ketorolac group compared to the control group	NA	NA	NA
Chiaretti [10]	NA	Remifentanil	Remifentanil 0.25 Ìg/kg/min	Rf was delivered at 0.25 μg/kg/min via continuous infusion, 1 h after admission to the pediatric intensive care unit (PICU). The treatment was continued for 12 h postoperatively.	1 episode of urinary retention	NA	NA	NA
Warren [31]	NA	Morphine	Morphine 10 to 40 μg/kg/h	10 to 40 μg/kg/h on a continuous morphine infusion order form. The infusion was titrated by the nurses within the rate parameters, based on the patient’s level of pain.	N/V	NA	NA	NA
Xu [2]	Posterior cranial vault expansion	Morphine, dexmedetomidine, acetaminophen	For patient 1: 1 mg morphine, dexmedetomidine 0.5 mcg/kg/h, acetaminophen 75 mg Q6H For patient 2: dexmedetomidine 0.2 mcg/kg/h, IV acetaminophen 86 mg Q6H, and morphine 0.4 mg Q3H PRN.	No clear protocol due to the study design	None	NA	NA	NA
Zubovic [33]	Endoscopic repair & open cranial vault remodeling	Acetaminophen,acetaminophen & ibuprofen, oxycodone	Oxycodone 5 mg/5 mL	Oxycodone 5 mg/5 mL suspension was the only opioid prescribed at discharge. The most common dosing applied was 0.05 mg/kg	NA	NA	NA	NA
Marel [28]	NA	Acetaminophen	Orally 20 mg/kg, rectal 40 mg/kg	Patients received 20 mg/kg acetaminophen either orally (*n* = 20) or rectally (*n* = 20) every 6 h after a rectal loading dose (40 mg/kg)	NA	NA	NA	NA

*NA* Not available, *LOS* length of hospital stay

### The postoperative pain scales

Twelve of the 16 articles included mentioned the postoperative pain assessment scale. The 10-point Face, Legs, Activity, Cry, Consolability (FLACC) Behavioral Pain Scale was utilized in six articles. Kattail et al. used the Wong-Baker Face pain scale, the 0–10 numerical rating scale score, and the FLACC scale. One study used the objective pain scale (OPS). Children’s Hospital of Eastern Ontario Pain Score (CHEOPS) was used in two studies. In one study, the Children and Infants Postoperative Pain Scale (CHIPPS) score was used, and 3 studies used Comfort-B. In one included study, visual analog scales were used. Four studies did not mention the pain assessment score.

### Complications related to the intervention

A total of seven studies reported postoperative complications. Nausea and vomiting were the main complications, reported in five studies [14, 30, 31, 35, 36]. Two studies observed a decline in hemoglobin levels, hematocrit levels, and blood loss [29, 37]. According to Tuncer et al., the ketorolac group had a lower postoperative hemoglobin than the control group [37]. There was one episode of urinary retention in the article by Chiaretti et al. [10]. Furthermore, Bronco et al.’s study was complicated by the emergence of delirium, sedation, respiratory depression, nausea, and vomiting [30]. There were three studies without complications [2, 38, 39].

### Length of hospital stay and follow-up

The length of follow-up visits after surgery was not mentioned in any of the articles. Eight studies, however, reported the length of the hospital stay. In the study conducted by Reddy et al., the group that did not receive dexmedetomidine stayed for 4.2 ± 1.0 days, while the group that received dexmedetomidine stayed for 4.0 ± 0.8 days [32]. According to a study by Tuncer et al., the hospital stay for patients receiving Ketorolac postoperatively is 2.1 days for those receiving Ketorolac compared to 2.6 days for those receiving a control dose [37]. According to Festa et al., the length of PICU stay for the scalp block group was 21.1 days, and for the control group was 18.1 days [39]. Knackstedt et al. found that the group following the ERAS protocol had a shorter hospital stay than the group not following it (ERAS group: 2.3 days, control group: 3.6 days) [34]. According to Fearon et al., the average hospital stay was two days [35]. Arts et al. found that hospitalization lasted 2.6 days [29], Kattail et al. found that it lasted 3.7 days [14], and Zubovic et al. found that it lasted one day [33].

### Parental satisfaction

There was only one study that reported parental satisfaction. A study by Festa et al. found that parental satisfaction levels were similar for both groups (Scalp block versus control group) [39].

### Quantitative data analysis

Meta-analysis was not possible due to the heterogeneity of the included articles.

### Identifying biases, quality assessment, and level of evidence

**Fig. 4:**
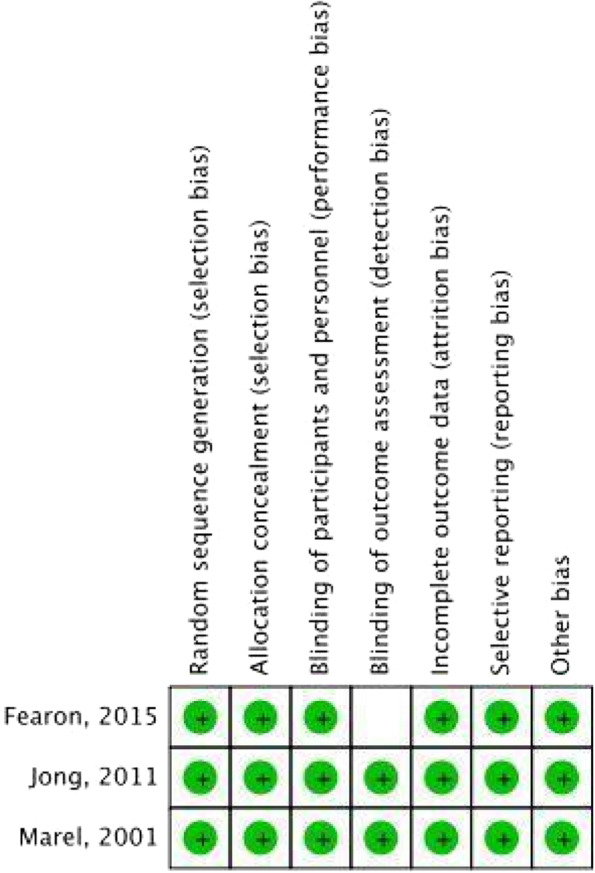
Risk of bias assessment summary for the randomized controlled trials

All included studies were evaluated based on the methodology of these studies. The bias risk was assessed separately and concurrently by two reviewers for the case series studies. The methodological quality and synthesis of the case series and case report was used, and the assessment tool is divided into four domains: selection, ascertainment, causation, and reporting (Table 4) [25]. The risk of bias assessment of eligible RCTs was done independently by two reviewers using the Cochrane Risk of Bias Assessment Tool for Randomized Trials (RoB 2). All of the three included RCTs were considered low risk of bias by the Revised Cochrane tool (Fig. 4) [26]. A Newcastle Ottawa Scale was used for the retrospective and prospective cohort studies. According to the Newcastle–Ottawa scale, case–control and cohort studies scored 7 out of 9, indicating a high quality (Table 5) [24]. According to the level of evidence and grading recommendations of the American Society of Plastic Surgery, two of the articles were level I, eleven articles level II, two articles level III, and one article level IV (Table 1) [23] (Table 6).

**Table 4:** A qualitative assessment of the case series included

**Domain For Evaluating the Methodological Quality of Case Reports and Case Series**								
**Reference**	**Selection**	**Ascertainment**		**Causality**		**Reporting**		
	**Leading Explanatory Questions**							
	**Q. 1**	**Q. 2**	**Q. 3**	**Q. 4**	**Q. 5**	**Q. 6**	**Q. 7**	**Q. 8**
Xu [2]	Yes	Yes	Yes	Yes	No	No	No	Yes

Selection: [question 1]. Does the patient(s) represent(s) the whole experience of the investigator (center) or is the selection method unclear to the extent that other patients with similar presentations may not have been reported?

Ascertainment: [question 2]. Was the exposure adequately ascertained? [question 3]. Was the outcome adequately ascertained?

Causality: [question 4]. Were other alternative causes that may explain the observation ruled out? [question 5]. Was there a challenge/rechallenge phenomenon? [question 6]. Was there a dose–response effect? [question 7]. Was follow-up long enough for outcomes to occur?

Reporting: [question 8] Is the case(s) described with sufficient details to allow other investigators to replicate the research or to allow practitioners to make inferences related to their own practice?

**Table 5:** The Newcastle–Ottawa Scale for the included cohort studies

Article	Cohort Studies								
	Selection				Comparability	Outcome			Quality Score
	Q1	Q2	Q3	Q4	Q5	Q6	Q7	Q8	
Arts [29]	*		*	*		*	*	*	
Reddy [32]	*	*	*	*	*	*	*	*	Good
Kattail [14]	*		*	*		*	*	*	
Tuncer [37]	*	*	*	*	*	*	*	*	Good
Knackstedt [34]	*	*	*	*	*	*	*	*	Good
Chiaretti [10]	*	*	*	*	*	*			Good
Warren [31]	*	*	*	*	*	*			Good
Bracho [36]	*	*	*	*	*	*			Good
Bronco [30]	*	*	*	*	*	*			Good
Zubovic [33]	*	*	*	*	*	*			Good

Selection: Q1. Representativeness of the exposure cohort? Q2. Selection of the non-exposure cohort? Q3. Ascertainment of exposure? Q4. Demonstration that outcome of interest was not present at start of the study?

Comparability: Q5. Comparability of cohort on the basis of the design or analysis?

Outcome: Q6. Assessment of outcome? Q7. Was follow-up long enough for outcomes to occure? Q8. Adequacy of follow-up of cohorts?

**Table 6:** The Newcastle–Ottawa Scale for the included case–control studies

Article	Case–Control Studies								
	Selection				Comparability	Exposure			Quality Score
	Q1	Q2	Q3	Q4	Q5	Q6	Q7	Q8	
Cercueil [38]					*				
Festa [39]	*	*	*	*	*	*			

Selection: Q1. Is the case definition adequate? Q2. Representativeness of the cases? Q3. Selection of controls? Q4. Definition of controls?

Comparability: Q5. Comparability of cases and controls on the basis of the design or analysis?

Outcome: Q6. Ascertainment of exposure? Q7. The same method of ascertainment for case and controls? Q8. Non-response rate?

## Discussion

Postoperative analgesia following open craniosynostosis repair is considered a challenge among plastic and reconstructive surgeons [15]. There is a persistent problem with pediatric patients suffering from acute postsurgical pain that is poorly treated [40–42]. Although numerous studies describe the etiology, evaluation, and treatment of craniosynostosis, few describe its pain management, even though some studies indicate a high prevalence of moderate to severe pain postoperatively [6]. In this systematic review, we compared the literature on perioperative pain management regarding potential clinical outcomes, recommendations, administration methods, and outcomes for different options for managing pain following craniosynostosis surgery.

A substantial amount of variability has been observed in the published data on intraoperative analgesia for craniosynostosis surgery. Among the seven studies, we found describing intraoperative pain management, each used a different protocol, from IV opioids alone to IV opioids combined with other drugs (e.g., Acetaminophen, NSAIDs, Gabapentin, and Dexmedetomidine). Thus, a unified intraoperative pain management protocol should be established through more studies in the future. As for postoperative analgesia, most studies used multimodal analgesia, with opioids (e.g., Morphine, Tramadol) and Acetaminophen being the most commonly used.

The known side effects of opioids range from nausea, vomiting, and urinary retention to more serious adverse effects such as respiratory depression, oversedation, and hypotension [10]. Dexmedetomidine has been used in some studies as a substitute for opioids to minimize these effects. A study by Reddy et al. in which the author describes using Dexmedetomidine as an opiate-sparing agent revealed that Dexmedetomidine was not associated with reduced opioid requirements by children postoperatively. The study also compared postoperative acetaminophen requirements, in which it found no significant difference between the group that received Dexmedetomidine versus the control group. However, patients who received Dexmedetomidine intraoperatively showed a significant reduction in their need for rescue medication for nausea and vomiting postoperatively [32]. Nonetheless, Fearon et al. pointed out that despite opioid avoidance, some craniosynostosis patients in their center who were given oral non-narcotics still suffered from nausea and vomiting [35]. Regarding respiratory depression and oversedation, the few reports that describe their occurrence in craniosynostosis patients treated with IV opioids suggest that these major complications are unlikely to occur [14, 31, 43].

In cranioplasty procedures, scalp nerve blocks (SNBs) have been reported to be adjuncts to traditional postoperative analgesia and as interventions for reducing intraoperative blood loss [38]. Guilfoyle et al.’s systematic review and meta-analysis found reduced postoperative pain when using regional SNBs in pediatric patients undergoing craniotomy [44]. However, current studies showed that the duration of postoperative opiate use following SNBs has not been found to be reduced [45]. Remifentanil is a potent synthetic opioid with a marked postoperative analgesic effect. Chiaretti et al. found that children who had a postoperative infusion of Remifentanil showed improvement in hemodynamic and behavioral parameters and pain control with no significant side effects, apart from one case of urinary retention. As a result, the children required further analgesia [12].

Furthermore, regarding the length of stay (LOS), one study demonstrated that the total doses of opioids administered postoperatively was not associated with the overall LOS [6]. On the other hand, Festa et al. found that adding SNB to the anesthetic protocol could potentially decrease the overall LOS compared to using general anesthesia alone [39]. However, LOS has not been explored in further depth. Therefore, more studies should explore the effect of various anesthetic and analgesic protocols on the length of stay in the field of craniosynostosis.

### Strength and limitations

This review has several limitations. Due to the heterogeneity of the included studies, no conclusions could have been drawn in the aggregate. In addition, meta-analysis was not possible. Also, the lack of consistency in the used pain medications, as well as their dosage, route of administration, and outcomes measured by the studies, prevents the development of substantial quantitative conclusions. Moreover, there is a scarcity in the available high-quality body of literature that looks into the pre-, peri-, and postoperative management of craniosynostosis. However, to the authors’ knowledge, this is the only systematic review that summarizes the use of analgesic agents in the pre-, intra-, and post-craniosynostosis repair surgery in the area. As part of our review, we focused on highlighting the fact that perioperative pain control for pediatric craniosynostosis patients is variable. In our study, the importance lies primarily in the usefulness of the tables and graphs used to report the different perioperative pain management options and the protocols for their application in clinical practice. Further comparative randomized controlled trials are required to determine the benefits and side effects of each agent. By comparing the intervention to the golden standard of care and to other interventions as well, we will be able to draw better, more accurate conclusions. For the management of postoperative pain after craniosynostosis surgery, standardized trials with clear, consistent, and non-biased outcomes can facilitate meta-analyses. To reduce the methodology disparity and improve the validity of the article by adding meta-analysis, we recommend that future studies focus mostly on prospective studies and RCTs. Studies are needed to compare the advantages and disadvantages of analgesia accurately. Also, future high-quality studies with large sample sizes are encouraged to establish a standard protocol for craniosynostosis perioperative pain management.

## Conclusion

The perioperative pain management plan is essential for any surgeon to decide prior to any major procedure, especially for pediatric patients undergoing invasive procedures such as craniosynostosis repair, which requires special considerations and regular adjustments. Based on this systematic review, the authors identified the most commonly used analgesics for pain control in pediatric patients undergoing cranioplasty, along with common side effects, length of hospitalization, and postoperative pain scores. Morphine is the most commonly used opioid as a single treatment, in combination with NSAIDs or acetaminophen. According to the results of this systematic review, the authors suggest the following: first, the use of opioids in combination with ketorolac, as it is found to have the shortest length of hospitalization and the lowest dose of opioids to control the pain. Second, SNB should be added to the intraoperative regimen as it is found to influence the length of hospitalization as well. Future clinical trials comparing the different types of analgesic combinations are recommended to further advance the understanding and practice of craniosynostosis pain management.

## Data Availability

The datasets used and/or analyzed during the current study are available from the corresponding author upon reasonable request.

## Abbreviations

NSAIDs: Nonsteroidal anti-inflammatory drugs
PICUs: Pediatric intensive care units
IV: Intravenous
PRISMA: Preferred Reporting Items for Systematic Reviews and Meta-Analyses
PROSPERO: Prospective Register of Systematic Reviews
CHEOPS: Children’s Hospital of Eastern Ontario Pain Score
FLACC: Face, Legs, Activity, Cry, Consolability
OPS: Objective pain scale
CC: Case–control
CS: Case series
R: Retrospective cohort
P: Prospective cohort
M: Male
F: Female
NA: Not available
USA: United States of America
N/V: Nausea/vomiting
SNB: Scalp nerve block
PCA: Parent/patient-controlled analgesia
LOS: Length of stay
